# Approaches to Enhancing Antioxidant Defense in Plants

**DOI:** 10.3390/antiox11050925

**Published:** 2022-05-08

**Authors:** Masayuki Fujita, Mirza Hasanuzzaman

**Affiliations:** 1 Laboratory of Plant Stress Responses, Faculty of Agriculture, Kagawa University, Miki-cho, Kita-gun, Takamatsu 761-0795, Japan; fujita@ag.kagawa-u.ac.jp; 2 Department of Agronomy, Faculty of Agriculture, Sher-e-Bangla Agricultural University, Dhaka 1207, Bangladesh

In the era of global climate change, plants are exposed to various adversities in field conditions [1]. Water stress (drought and waterlogging), salinity, metal/metalloid toxicity, extreme temperatures, xenobiotics, and other abiotic stressors have a significant impact on plant growth, development, and sustainable crop production [2]. Different reactive oxygen species (ROS) including free radicals, (superoxide anion, O_2_^•−^; hydroperoxyl radical, HO_2_^•^; alkoxy radical, RO^•^; and hydroxyl radical, ^•^OH) and non-radical molecules (hydrogen peroxide, H_2_O_2,_ and singlet oxygen, ^1^ O_2_) are naturally produced in plants as a result of the cellular metabolism [2,3,4]. However, the overproduction of ROS occurs in plant cells under stress. ROS are highly reactive, interfering with plant metabolism and causing significant damage to essential cellular components such as carbohydrates, lipids, proteins, DNA, and others [5]. Therefore, this disrupts the balance between normal ROS generation and antioxidant activity, leading to oxidative stress in plants [2].

Enhancing the capacity of the antioxidant defense system in plants is the main adaptive response to oxidative stress [6]. The system is mainly maintained by some low-molecular-weight nonenzymatic antioxidants, such as ascorbic acid (AsA), glutathione (GSH), α-tocopherol, phenolic compounds, flavonoids, alkaloids, and nonprotein amino acids along with other antioxidant enzymes [2]. Likewise, a bunch of antioxidant enzymes are associated with this defense system, including peroxidase (POD), superoxide dismutase (SOD), catalase (CAT), ascorbate peroxidase (APX), glutathione reductase (GR), monodehydroascorbate reductase (MDHAR), dehydroascorbate reductase (DHAR), glutathione peroxidase (GPX) and glutathione *S*-transferase (GST) [7]. For example, SOD removes O_2_^•−^, CAT converts H_2_O_2_ into H_2_O and O_2_, POD scavenges H_2_O_2_ in the vacuoles, GST combines GSH with the electrophilic or hydrophobic compounds, and MDHAR and DHAR control the ascorbate pool [8]. Other xenobiotics and electrophilic compounds are converted into less hazardous molecules by the GST and GPX enzymes and eventually sequestered in extracellular spaces [9]. One of the vital defense systems is the AsA-GSH cycle, which regulates the levels of H_2_O_2_ [4]. Żur et al. [10] reported that microspore embryogenesis in triticale was induced through various stress treatments of tillers, and its effectiveness was analyzed in terms of AsA and GSH contents, the total activity of low-molecular-weight antioxidants, and the activities of AsA-GSH cycle enzymes. The exogenous application of melatonin (MEL) was found to be effective in enhancing plant defense under a low temperature [10].

The importance of an antioxidant defense system in plants is crucial for the plants in stressful environments as it delays programmed cell death. In the absence of sufficient antioxidant enzymes in plants to scavenge excessive ROS, cellular organelles cannot properly continue their activities, resulting in lipid peroxidation, protein damage caused by oxidation, breakdown of DNA molecules and nucleic acids, and several enzyme inhibitions [11]. The antioxidant defense system ensures efficient ROS detoxification, reduced lipid peroxidation in membranes, and the prevention of protein damage by delaying oxidation and controlling DNA and nucleic acid damages under stress. Thus, by providing overall cellular protection, this enables the plants’ stress tolerance abilities.

However, antioxidant defense efficiency differs between plant species and genotypes, as well as between various stress conditions and their frequencies [2]. El-Badri et al. [12] found that the ROS level and malondialdehyde (MDA) content were minimized in tolerant genotypes due to the activation of antioxidant enzymes, such as SOD, POD, CAT, and APX to scavenge over-accumulated ROS under salinity stress. Most of the contributors to stress tolerance are relevant to amino acids, sucrose, flavonoid metabolism, and tricarboxylic acid cycle, which accumulated as a response to salinity stress. The tolerant cultivar showed improved antioxidant enzyme activity and higher metabolite accumulation, which enhances its tolerance against salinity. Their study is of great reference value for plant breeders to develop salt-tolerant rapeseed cultivars [12]. Begum et al. [13] investigated the antioxidant metabolism in four soybean cultivars, viz., PI408105A, PI567731, PI567690, and PI416937 exposed to drought (5, 10, and 15% polyethylene glycol, PEG-6000), salinity (50, 100, and 150 mM NaCl), and their combination, particularly at the seed germination stage. They found that the ROS accumulation was accompanied by improved enzymatic antioxidant activity, such as SOD, POD, CAT, and APX. However, the enhancement was most noticeable in PI31 and PI90 under both salinity and drought conditions. They concluded that the stress tolerance and improved seed germination of soybean are mainly regulated by its superior antioxidative enzyme activity and secondary metabolites [13].

The production of ROS in plant cells displays both detrimental and beneficial effects. However, the exact pathways of ROS-mediated stress alleviation have yet to be fully elucidated. Sachdev et al. [14] summarized the status of known production sites, signaling mechanisms/pathways, effects, and the management of ROS within plant cells under stress. Moreover, they discussed the role played by advancements in modern techniques such as molecular priming, systems biology, phenomics, and crop modeling in preventing oxidative stress, as well as diverting ROS into signaling pathways [14]. Cross-tolerance is one of the plant adaptive responses to abiotic stress associated with ROS signaling. Rani et al. [15] reported that cold acclimation remarkably enhanced enzymatic (SOD, CAT, APX, GR) and non-enzymatic (AsA and GSH) activity, which resulted in reduced low-temperature-induced leaf damage under cold stress in tolerant chickpea genotypes. This information will be useful in directing efforts to increase cold tolerance [15].

Phytohormones have a significant function in stress signaling and oxidative stress mitigation, along with their direct involvement in growth control. The use of probiotic bacteria from the genera *Bacillus*, *Pseudomonas*, *Enterobacter*, *Micrococcus*, *Lysobacter*, and others improves plant tolerance to a variety of stressors [6]. Qadir et al. [16] revealed that phytohormone-producing, plant-growth-promoting rhizobacteria *Acinetobacter bouvetii* P1 restored the sunflower growth under Cr^+6^ by strengthening the host antioxidant system and triggering the higher production of enzymatic antioxidants, including CAT, APX, SOD, and POD. Moreover, P1 also promoted a higher production of nonenzymatic antioxidants, such as flavonoids, phenolics, proline (Pro), and GSH [16]. Fatma et al. [6] found that exogenous application of methyl jasmonate (MeJA) resulted in an increased enzymatic antioxidant activity that reduced the H_2_O_2_ content and thiobarbituric acid reactive substances and enhanced the photosynthetic efficiency of wheat under heat stress (42 °C).

The addition of signaling molecules such as nitric oxide (NO), hydrogen sulfide (H_2_S), and H_2_O_2_ stimulates the antioxidant system. Some stress-tolerant plant species and halophytes showed a better ability to synthesize signaling molecules such as NO, which is correlated with stress tolerance. Hasanuzzaman et al. [17] observed that the enhanced salt tolerance in halophyte *Kandelia obovata* was strongly associated with the antioxidant defense, which was governed by NO. The treatment of salt-stressed plants with sodium nitroprusside (SNP) increased endogenous NO levels, reduced ion toxicity, and improved nutrient homeostasis while further increasing Pro levels. SNP treatment also improved the activities of antioxidant enzymes (CAT, APX, MDHAR, DHAR). However, treatment with NO scavengers or inhibitors reversed these beneficial SNP effects and exacerbated salt damage, confirming that SNP promoted stress recovery and improved plant growth under salt stress [17]. Rahman et al. [18] observed that the NO donor could sustain alfalfa plants from iron (Fe) deficiencies. Exogenous NO donor restored Fe-homeostasis and oxidative status in Fe-deficient alfalfa. Specifically, the increase in antioxidant genes and their related enzymes (Fe-SOD, APX) in response to SNP treatment suggests that Fe-SOD and APX are key contributors to reductions in H_2_O_2_ accumulation and oxidative stress in alfalfa. Furthermore, the elevation of AsA-GSH pathway-related genes (GR and MDAR) in Fe-deficiency with SNP implies that the presence of NO relates to an enhanced antioxidant defense against Fe-deficiency stress [18].

Recently, MEL has been widely tested in abiotic stress situations including drought, waterlogging, and salinity [19]. In their review article, Moustafa-Farag et al. [19] summarized that MEL controls the ROS levels and reactive nitrogen species, and positively changes the molecular defense to improve plant tolerance against water stress. Moreover, the crosstalk between MEL and other phytohormones is a key element of plant survival under drought stress, while this relationship needs further investigation under waterlogging stress [19].

Qi et al. [20] revealed that improvements in endogenous GABA levels in leaf and root by GABA pretreatment could significantly alleviate the damage to white clover during high-temperature stress. The GABA significantly enhanced the gene expression and enzyme activities involved in antioxidant defense, including SOD, CAT, POD, and key enzymes of the AsA–GSH cycle, thus reducing the accumulation of ROS and the oxidative injury to membrane lipids and proteins. In addition, the expression and decline in the GABA-induced aquaporin in endogenous abscisic acid levels could improve the heat dissipation capacity by maintaining a higher stomatal opening and transpiration in white clovers under high-temperature stress [20].

Phytohormone-based seed priming also proved to be effective against abiotic stress. Basit et al. [21] concluded that seed priming with brassinosteroids (EBL) could be adopted as a promising strategy to enhance rice growth by copping the toxic effect of chromium (Cr). They [21] revealed that seed priming with a low dose (0.01 µM) of EBL could alleviate the adverse effects of Cr in two different rice cultivars. Seed priming with EBL stimulatingly increased antioxidant enzyme activities to scavenge ROS production under Cr stress. The gene expression of SOD and POD in EBL-primed rice plants followed a similar increasing trend, as observed in the case of enzymatic activities of SOD and POD compared to water-primed rice plants. Simultaneously, Cr uptake was observed to be higher in the water-primed control compared to plants primed with EBL.

Antioxidant metabolism has been improved by a variety of plant nutrients [8]. According to Imran et al. [22], molybdenum (Mo) supply was found to strengthen plant metabolism at prominent growth stages through an improved enzymatic and non-enzymatic antioxidant defense system, thereby increasing the grain yield and quality characteristics of aromatic rice under cadmium (Cd) toxicity. Importantly, Mo supply enhanced photosynthesis, Pro, and soluble protein content, and also strengthened plant metabolism and antioxidant defense by maintaining higher activities and transcript abundance of ROS-detoxifying enzymes at the vegetative, reproductive, and maturity stages of aromatic rice plants under Cd toxicity [22].

Researchers have used a variety of techniques to reduce the negative consequences of oxidative damage by increasing antioxidant defenses [6]. Approaches ranging from genetic manipulation to the introduction of exogenous protectants in plants. Characterization and profiling antioxidant enzymes are among the targeted approaches. In their study, Su et al. [23] performed a genome-wide investigation to identify the rapeseed *SOD* genes. They recognized 31 *BnSOD* genes in the rapeseed genome, including 14 *BnCSDs*, 11 *BnFSDs*, and six *BnMSDs*. In brief, gene ontology annotation outcomes confirm the *BnSODs* role under different stress stimuli, cellular oxidant detoxification processes, metal ion binding activities, SOD activity, and different cellular components. Moreover, the expression profiling showed that eight genes (*BnCSD1*, *BnCSD3*, *BnCSD14*, *BnFSD4*, *BnFSD5*, *BnFSD6*, *BnMSD2*, and *BnMSD10*) were significantly up-regulated under different hormones (abscisic acid, gibberellic acid, indole acetic acid, and kinetin) and abiotic stress (salinity, cold, waterlogging, and drought) treatments. Their findings deliver the foundation for additional functional investigations into the *BnSOD* genes in rapeseed breeding programs. Rudíc et al. [24] have analyzed eight functional *SOD* genes from potato, three *StCuZnSODs*, one *StMnSOD*, and four *StFeSODs*. The quantitative analysis revealed a higher induction of *StCuZnSODs* (the major potato *SODs*) and *StFeSOD3* in thermotolerant cultivars than in thermosensitive cultivars during long-term exposure to elevated temperature. *StMnSOD* was constitutively expressed, while the expression of *StFeSODs* was cultivar-dependent. Their results provide the basis for further research on *StSODs* and their regulation in potato, particularly in response to elevated temperatures [24].

This Special Issue, “Approaches in Enhancing Antioxidant Defense in Plants” published 13 original research works and a couple of review articles that discuss the various aspects of plant oxidative stress biology and ROS metabolism, as well as the physiological mechanisms and approaches to enhancing antioxidant defense and mitigating oxidative stress. These papers will serve as a foundation for plant oxidative stress tolerance and, in the long term, provide further research directions in the development of crop plants’ tolerance to abiotic stress in the era of climate change.
